# Dental Hints Department

**Published:** 1907-02

**Authors:** B. H. Teague

**Affiliations:** Aiken, S. C.


### ...DENTAL HINTS DEPARTMENT...

CONDUCTED BY B. H. TEAGUE, D. D. S., AIKEN, S. C., TO WHOM ALL CONTRIBUTIONS TO THIS DEPARTMENT SHOULD BE SENT

Remove all enamel rods that are not supported by dentine.—Dr. C. E. Woodbury, *Review*.

Iodoform.—Has it any virtue that may not be found in higher degree in several other agents that are innocent of its incomparable odor?—Dr. H. Clark, *Dom. Journal*.

Exposure of cement liquid to the moist atmosphere of summer time results in the taking up of water; exposure to the dry atmosphere of the average office in midwinter results in the giving up of water.—Dr. W. V. B. Ames, *Era*.

To remove calcarous deposits from rubber plates, drop them in a solution of muriatic acid and water; one part of acid to ten of water. Result is immediate.—*Western Dental Journal*.

The Garhart Dental Manufacturing Company Sells Out.—The Harvard Company. of Canton, Ohio, has purchased the plant of the Garhart Dental Manufacturing Company, of Indianapolis, Ind., and will incorporate that company into the Harvard.

The cavo-surface angle should be beveled from one-fifth to two-thirds the thickness of enamel, depending upon the location of the cavity wall and condition and direction of the enamel rods.—Dr. C. E. Woodbury, *Review*.

I cannot consistently give definite examples of cements for special purposes, but the selections are easy, since the cement for a tropical atmosphere must be slow of setting, while in a cool, dry atmosphere such an one would prove unsatisfactory.—Dr. W. V. B. Ames, *Era*.

Few who know Dr. E. C. Kirk would rate him a profane man, and yet a contributor to a recent *Cosmos* once heard him say: "If a tooth is worth a dam, put it on."—*Office and Laboratory.*

FORMALDEHYDE.—Specific value lies in the fact that its high germicidal power is due to a gas in solution, and this gas penetrates and permeates where a fluid will not. Its specific objection is in the intense irritation imparted to sensitive tissues.—DR. H. CLARK, *Dom. Journal.*

Extend gingival margins under the free margin of the gum and all other margins to smooth surfaces in such a position that they will be kept clean by the excursions of food and the action of the lips, cheeks and tongue.—DR. C. E. WOODBURY, *Review.*

TREATING CAVITIES WITH NITRATE OF SILVER.—I believe it is a good practice when you have some evidence of a superficial decay to touch it with nitrate of silver and see how deep it will go. In a few weeks that will show you how far the area of decay has gone. Lots of times you need not go any further, as that will stop the decay.—J. N. CROUSE, *Dental Digest.*

AFTER-PAINS OF EXTRACTION.—Relief may be given in a short time by inserting in the socket a pellet of absorbent cotton dipped in chloroform; place in each root socket, leaving it there a minute or two. In extreme cases repeat. Relief is sure to follow.—H. A. CROSS, *Dental Review.*

I have always had misgivings as to whether it is altogether right to foster too much presumption of ability on the part of the dental graduate to administer anesthetics. I hold that we should teach that the dental graduate should never administer chloroform or ether.—DR. A. G. FRIEDRICHS, *Dom. Journal.*

THE USE OF TALC SUPPORTS IN THE FUSING OF PORCELAIN.—"Talc," the commercial form of boiler makers' crayons, obtainable in heavy hardware stores, is useful as supports for porcelain work in the furnace. It may be carved to any form desired, and withstands heat perfectly.—GOODMAN A. MILLER, *The Bur.*

It is rather doubtful if any court in the land would exonerate a man from blame, not to say criminal carelessness, for fatal termniation of a case following the use of a local anesthetic, the ingredients of which he knew nothing of.—DR. R. T. OHNER, *Dom. Journal.*

THE RUBBER DAM.—New rubber dams should be thoroughly washed with soap and water; or, better still, boiled before using. Any one who has not practiced this will be astonished at the amount of impurity removed in this process; it certainly offends against the canon of good taste to allow the patient to suck this off, though there may be no question of any specific disease germ.—J. H. BADCOCK, *British Dental Journal.*

DENTAL ETHICS.—Training in ethics must begin in the kindergarten and primary school; it must have the continuous influence of the home in its development, and must be continued throughout the preparatory period. Lacking this background the giving of a few lectures upon dental ethics is wasted. No student can understand dental ethics if he has no ethical sense to which to appeal.—E. C. KIRK, *Dental Cosmos.*

TO POLISH A PORCELAIN CROWN OR TOOTH THAT HAS BEEN GROUND.—Make a very soft paste of a saturated solution of spirits of turpentine, gum camphor and pumice flour. Keep your polishing wheel wet with this mixture while repolishing the tooth. You will be pleased with the result.—W. H. SPAULDING, *Dental Summary.*

OFFICERS OF THE NATIONAL DENTAL ASSOCIATION.—President, Dr. A. H. Peck, Chicago; vice president for the East, Dr. Geo. E. Hunt, Indianapolis; vice president for the South, Dr. Geo. Van, of Gadsden, Ala. vice president for the West, Dr. D. J. McMillan, Kansas City, Mo.; recording secretary, Dr. Chas. S. Butler, Buffola; corresponding secretary, Dr. Burton Lee Thorpe, St. Louis; treasurer, O. R. Mellindy, Knoxville; executive committee, C. M. Work, York, and T. P. Hinman, Atlanta.

When dental plaster of a good quality and water are mixed in proper proportions, re-crystallization or setting usually begins in from two to six minutes. At the initial stage of the setting process a very slight amount of contraction is noticeable; then follows a short period of inertia lasting a minute or two, after which expansion sets in.—Dr. J. H. Prothero, *Register*.

Setting of Cement.—The state of division of a powder affects the setting with a given liquid; the finer the powder the quicker the setting. This is simply the result of the greater surface presented for chemical action. Experiments conducted some years since indicate that the strength of cement is enhanced by extra fineness of powder up to a certain point, but that absolute lack of grit causes a falling off in strength, the greatest strength being from a powder presenting a very slight evidence of grain.—Dr. W. V. B. Ames, *Era*.

Silicate Cement Fillings.—I have noticed that the fillings which have loosened from shrinkage have been in position where it was most difficult to apply pressure. I believe that the granular breaking away that sometimes occurs around the edge of the filling is caused by the addition of the powder to the mixture after crystallization has begun. The lack of translucency in these failures shows incomplete assimilation of the powder.—Dr. C. L. Brimstool, *Office and Laboratory*.

Cement Mix.—To best open this subject, we will assume that it is conceded that there is a certain desirable consistency to which an oxyphosphate of zinc cement should be mixed for filling purposes and another consistency most desirable for the setting of crowns, bridges and inlays. There is a certain homogeneous condition—stiff—best for filling, and for crowns and inlays cement should be as stiff as will admit of the piece going readily to place.—Dr. W. V. B. Ames, *Era*.

Sight of the Infant.—The infant is incapable of binocular vision, that is to say, single vision with the two eyes is not accomplished by most children until the third month, in some cases not until the sixth month, and occasionally not until the child is a year old. Again, if one is far-sighted or astigmatic (and most of us present one or both of these froms of refraction) the attempt to see distinctly with both eyes at close range is still more productive of eye strain.—Dr. C. A. Wood, *Review*.

Annealing Gold.—I have a suggestion to offer regarding annealing. In offices in which the alcohol flame is to be used, if gold is kept in ammonia fumes, and thus kept from contamination with other gases (an ammonia salt is formed, and that ammonia salt is the most volatile of all common salts), the gold is more easily annnealed. The salts or gases that are on the so-called non-cohesive golds are not as easily driven off.—*Dental Digest*.

Silk First—Then the Clamps.—I desire to call attention to the application of one or two ligatures in placing a clamp upon a tooth. One circle of floss silk will prevent the clamp from impinging upon the gum. If the tooth is cone shaped, two should be put on; one will prevent the clamp from moving upward, and the other from jumping off, and there will be saved the pain from impingement.—Dr. N. C. Register, *Brief*.

Adapting Cement Fillings.—In filling with oxyphosphate of zinc the ideal adaptation of cement to cavity wall is secured by taking from the mix at the thick, creamy or crown-setting stage enough cement to line the cavity walls, the balance of the mix to be made as stiff as it can be properly spatulated. This proper stiffness of mix is indicated by the cement rolling up after the spatula instead of remaining upon the slab as a smooth film.—Dr. W. V. B. Ames, *Era*.

Spatulating Cement Fillings.—Most often cement does not receive sufficient careful spatulation, yet it can be utterly ruined by overspatulation. Too little spatulation gives a quick-setting, granular result, and overspatulation gives cement which will never properly crystallize. Thus it can be seen that these features must be kept in mind and that the operator must become familiar with the proper *feel* of the cement beneath the spatula.—Dr. W. V. B. Ames, *Era*.

Silver Nitrate.—For ten years I have made it a rule to see that all teeth back of the canines were given a good treatment with a saturated solution of silver nitrate as soon as possible after eruption. I apply it with a small swab, letting it stay a minute and pushing it with an explorer down into the sulci. The staining is only superficial and caries is generally prevented, and if it occurs it is greatly retarded.—H. F. HAMILTON, *International Dental Journal.*

REMEDY FOR PYORRHOEA.—The variety of remedies used in the treatment of pyorrhœa alveolaris has not changed much. Iodo-Glycerol, after all the deposits have been removed

℞

| | |
|---|---|
| Zinci Iodidi . . . . . . . . . . . | 15. |
| Tinct. Iodidi . . . . . . . . . . . | 25. |
| Glycerini . . . . . . . . . . . . | 50. |
| Aquae . . . . . . . . . . . . . | 10. |

and with daily treatments, has given promising results, although a 10 per cent solution of Iodide of Zinc alone is almost as efficient.—DR. E. T. LEFFLER, *Magazine.*

TREATMENT OF PUTRESCENT CANALS.—One of the most careful operators in this country, every time a patient presents with a putrescent pulp or abscess, whether blind or with a sinus, goes down into that canal and chemically sterilizes not only the canal, but the tubili as well. He does it by means of Schreier's paste, sodium and potassium, which unites chemically with the end products and the intermediate products of pulp decomposition.—DR. J. P. BUCKLEY, *Review.*

ANTISEPTIC AND ANESTHETIC PASTE.—White vaseline, 1 ounce; cocaine, 14 grains; menthol, 24 grains; oil of peppermint, 10 grains; chloretone, 9 grains; phenol, 2 grains. Mix and apply before scaling the teeth by rubbing it into the spaces between the teeth and on the gums.—*Dentist's Magazine.*

CRACKING OF TEETH IN SOLDERING.—Personally, it is years since I had such an accident happen, and I attribute my immunity from this to the following points: (1) The use of only the best makes of teeth. (2) No riveting when backing. (3) Care in applying borax around the pins. (4) Care in slowly heating up preparatory to soldering. (5) Soldering in one operation, so that there are no sudden changes of temperature. (6) Cooling down naturally.—B. L., *Quarterly Circular.*

Occlusion for Bridges.—In determining upon the occlusion of a bridge, we must remember that the life of the bridge is the life of its weakest anchorage. In a bridge supported by two teeth, one a multi-rooted and the other single-rooted, both in the same condition of health, if the forces of mastication bear equally on each tooth, the single-rooted one will loosen first, even if the force be not applied at an angle to its long axis, as is usually the case in single-rooted teeth. Hence it follows that if you favor that tooth at the expense of the other in the arrangement of occluding surfaces, you will have increased, to some extent, the life of the appliance.S. H. Voyies, *Dental Brief.*

Objection to Adrenalin Chlorid.—The well-known liability of secondary hemorrhage after the use of adrenalin renders it unsatisfactory as a menstruum for cocain in pressure anæsthesia. I recently used it in removing the pulp from a central incisor and packed the canal with cotton saturated with an anæsthetic. At the next sitting I found the entire crown of a decidedly pinkish hue, showing a thorough infiltration of the tooth substance with hæmaglobin. The vigorous use of pyrozone practically restored the tooth to its natural color, though not entirely so.—*Dental Brief.*

Danger in the Use of Anaesthetics.—There never has been a patient anæsthetized in this world to a state of complete anæsthesia who has not been placed in a dangerous condition. Every time an anæsthetic is administered, no matter what it is, the man who administers it is taking upon himself a great responsibility, and the possibility of the collapse and loss of the patient. I have lived with anæsthetics most of my life. I have administered them thousands of times, and yet the older I grow, and the more I do in the use of them, the more I feel the responsibility.—Dr. T. W. Brophy, *Dom. Journal.*

Neglect to Restore Contour.—It is true that for a few years during immunity from dental cares, it would seem that no amount of neglect or abuse would have any unfavorable effect on the teeth, but recollect that during that period and before the age of from 40 to 45 the teeth from abrasion of the contact points lose about 10 mm. of their total mesio-distal diameter. If we do not restore this in some way, there will be a total loss of contact and a recession of the gum tissue which protects the concavity of the tooth, leaving a nidus in which microbic plaques may attach themselves where without fear of being disturbed they may secrete the acid which will dissolve the lime salts of the tooth.—Dr. W. R. Clark, *Review*.

To Prevent Soreness.—In regard to after-soreness, Dr. Eschelman of Buffalo suggested something to me about a year ago which has proved very satisfactory in preventing it. It consists, after removing the pulp with pressure anesthesia and waiting for the hemorrhage to subside, in placing in the canal a dressing of cotton saturated with full-strength extract of witch hazel, leaving the dressing in the canal, dismissing the patient, and taking it out at the next sitting, at which time the root-canal is filled. Since I have followed this plan I have had very little trouble from after-soreness.—*Dental Cosmos*.

Cement Linings With Amalgam Fillings.—There are many cases where a lining of cement is desirable when amalgam is to follow, and especially where the loss of dentin leaves a section of translucent enamel, which would show blue with the amalgam immediately in contact. One very satisfactory way to proceed is to cover the cavity walls with cement, and then having amalgam mixed and ready, crowd it in in a way to force out the surplus cement, finally clearing the margins of cement, so that the finished filling shows no line of cement exposed. This might not inaptly be called an amalgam inlay, and would not be a bad idea to apply in nearly all cases where amalgam is to be used. If painstakingly done, the method is ideal. It should be done with some rapidity to be able to force out surplus cement, and especially at obscure margins before crystallization begins. —R. B. Tuller, *American Dental Journal*.

Treatment of Putrescent Pulps.—Adjust rubber dam and sterilize all included teeth, using either a ten per cent solution formaldehyde to which a small amount of borax has been added, or a 1:500 solution mercury bichlorid in cinnamon water. Then bathe the teeth in alcohol and open freely, exposing all the pulp canals, and place over the mouth of each a small pledget of cotton carrying equal quantities of cresol and formalin sealed in hermetically, preferably with a quick-setting cement. Dismiss the patient for two or three days, or a week if more convenient. At the second sitting remove the dressing, cleanse the canals mechanically and gently work down into each canal a 1:500 solution of mercury bichlorid in hydrogen dioxide; dehydrate with alcohol and warm air, and place in each canal cotton carrying 2 parts cresol and 1 part formalin. Seal in and leave for at least three days, when the remedy will have sterilized the entire tubular structure of the dentin, thus establishing asepsis, when the canals should be thoroughly filled.—J. P. Buckley, *Dental Cosmos.*

Eruption Caused by Mouth-Washes.—Within recent years dermatologists have called attention to a peculiar eczematous eruption about the external mouth, which is caused by a constant irritation of the affected parts from the use of mouth-washes containing a comparatively large amount of essential oils. Neisser, in 1898 and 1902, mentions two cases, and now Galewsky, of Dresden, reports sixteen additional cases in which mouth-washes containing these irritating essential oils were directly responsible for the causation of the disease. From our own observation we are able to increase the list by two more cases. This disease manifests itself as a peculiar scaly eruption about the lips and chin, but more especially at the corners of the mouth. According to Galewsky, those suffering from seborrhea or eczema are especially predisposed to this affection. Usually medication will be of no avail unless the primary cause, the irritation by the mouth-wash, is removed.—Dr. Herman Prinz, *Era.*

Soldering Teeth.—Although I constantly use American teeth, I have found those of English manufacture much more satisfactory for soldering purposes. Care should be taken to cover up all the porcelain with the investing compound, and to cut away as much of the investment as possible where it would interfere with the free play of the blowpipe flame. It goes without saying that heating up and cooling down must be done much more slowly when soldering teeth than when only soldering bands. For heating I allow about ten minutes with the gas very low, ten minutes more with it rather higher, and another ten minutes with it full on. I find this very satisfactory. I use a few small pieces of solder to make the actual joint, and then add large pieces to strengthen it and make the desired contour. After taking the piece from the stove, all the operations should be carried out quickly. In my judgment prolonged heating and alternate heating and cooling are responsible for many cracked teeth and crystallized pins. When soldering is done I place the dome over the work again and let it cool very slowly.—*The Dental Magazine.*

Dental Paste.—To those in the profession who are interested in the use of such remedies, I would advise a trial of the following. The liquid should consist of one part of formalin and three parts of cresol and the powder of 5 parts of thymol, 12 parts of dry alum and 40 parts of kaolin or silicate of alumina. This formula, if carefully prepared and intelligently used, will give much better results than any of the nostrums that have been so widely advertised. Kaolin is in many respects a better vehicle than oxide of zinc, because the latter will in time set and is, therefore, hard to remove should the case require it. These pastes are often recommended in the treatment of irritated apical tissue. This is, in many respects, the most difficult tissue to treat and the treatment varies with the cause and the degree of irritation. In the opinion of Dr. Inglis such tissue requires most careful handling. We need not resort to patented or proprietary pastes. Sedative preparations, such as tincture aconite with a little cocaine, or Campho phenique plus a small amount of menthol to add to its diffusibility are the remedies indicated.—Dr. E. T. Leffler, *Magazine.*

Local Anesthetics—As to local anesthetics—cocaine—I do not know much about it; I might say I do not know anything about it. I have never seen a man (and I have talked with many) who can tell you what the minimum dose is that may be used with safety. They do not know, because a patient will sometimes collapse when a fraction of one per cent is used. So I say, and if I am wrong I would be glad to be corrected, I do not know the man who can tell how little can be administered with safety. Therefore, I do not use it; I do not like to use a thing that I know nothing about. We are deluged with these proprietary remedies or anesthetics. It says in the journals, "May be used with safety," and our students are trained in a way to think they may use certain agents with safety. There is no absolute safety connected with the administration of any of them, and I wish I had the power of wiping out of the journals those advertisements set forth as sure cures, etc., because such statements are not true.—Dr. T. W. Brophy, *Dominion Journal.*

